# Sterol Regulatory Element-Binding Protein (Sre1) Promotes the Synthesis of Carotenoids and Sterols in *Xanthophyllomyces dendrorhous*

**DOI:** 10.3389/fmicb.2019.00586

**Published:** 2019-03-29

**Authors:** María Soledad Gutiérrez, Sebastián Campusano, Ana María González, Melissa Gómez, Salvador Barahona, Dionisia Sepúlveda, Peter J. Espenshade, María Fernández-Lobato, Marcelo Baeza, Víctor Cifuentes, Jennifer Alcaíno

**Affiliations:** ^1^Departamento de Ciencias Ecológicas, Facultad de Ciencias, Universidad de Chile, Santiago, Chile; ^2^Centro de Biotecnología, Facultad de Ciencias, Universidad de Chile, Santiago, Chile; ^3^Department of Cell Biology, Johns Hopkins University School of Medicine, Baltimore, MD, United States; ^4^Centro de Biologiìa Molecular Severo Ochoa, Departamento de Biologiìa Molecular (UAM-CSIC), Universidad Autoìnoma de Madrid, Madrid, Spain

**Keywords:** *X. dendrorhous*, SREBP/Sre1, carotenogenesis, astaxanthin, sterols, ergosterol, transcriptional regulation

## Abstract

*Xanthophyllomyces dendrorhous* is a basidiomycete yeast that synthesizes carotenoids, mainly astaxanthin, which are of great commercial interest. Currently, there are many unknown aspects related to regulatory mechanisms on the synthesis of carotenoids in this yeast. Our recent studies showed that changes in sterol levels and composition resulted in upregulation of genes in the mevalonate pathway required for the synthesis of carotenoid precursors, leading to increased production of these pigments. Sterol Regulatory Element-Binding Proteins (SREBP), called Sre1 in yeast, are conserved transcriptional regulators of sterol homeostasis and other cellular processes. Given the results linking sterols and carotenoids, we investigated the role of SREBP in sterol and carotenoid synthesis in *X.* *dendrorhous.* In this study, we present the identification and functional characterization of the *X.* *dendrorhous* *SRE1* gene, which encodes the transcription factor Sre1. The deduced protein has the characteristic features of SREBP/Sre1 and binds to consensus DNA sequences *in vitro.* RNA-seq analysis and chromatin-immunoprecipitation experiments showed that genes of the mevalonate pathway and ergosterol biosynthesis are directly regulated by Sre1. The *sre1^-^* mutation reduced sterol and carotenoid production in *X. dendrorhous*, and expression of the Sre1 N-terminal domain (Sre1N) increased carotenoid production more than twofold compared to wild-type. Overall, our results indicate that in *X. dendrorhous* transcriptional regulation of genes in the mevalonate pathway control production of the isoprenoid derivatives, carotenoids and sterol. Our results provide new insights into the conserved regulatory functions of SREBP/Sre1 and identify pointing to the SREBP pathway as a potential target to enhance carotenoid production in *X. dendrorhous*.

## Introduction

*Xanthophyllomyces dendrorhous* is a basidiomycete yeast that synthesizes carotenoids, mainly astaxanthin, pigment of great commercial interest widely used in aquaculture and in nutraceutical, pharmaceutical and cosmetics industry (Galasso et al., 2017). *X. dendrorhous* was originally isolated from tree exudates in mountainous regions in the northern hemisphere, such as Japan and Alaska by Phaff and co-workers in 1960 (Miller et al., 1976). Subsequently, isolates have been obtained from different regions around the world (Contreras et al., 2015).

**FIGURE 1:**
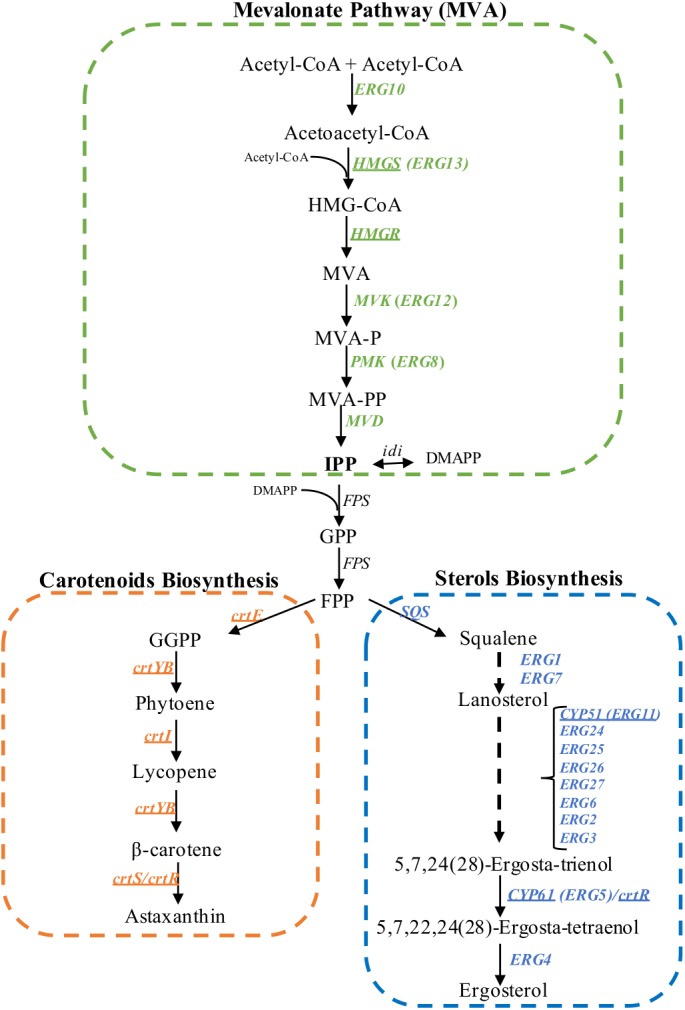
Biosynthesis of isoprenoids in *X. dendrorhous.* Mevalonate pathway (in green), astaxanthin (in orange) and ergosterol biosynthesis (in blue). The arrows represent catalytic steps and the respective enzyme encoding gene. Underline genes have been functionally characterized in *X. dendrorhous*. The ergosterol biosynthesis pathway was abbreviated based on the pathway described in *S. cerevisiae.* HMG-CoA, 3-hydroxy-3-methylglutaryl-CoA; MVA, mevalonate; MVA-P, mevalonate-5-phosphate; MVA-PP, mevalonate-5-pyrophosphate; IPP, isopentenyl-pyrophosphate; DMAPP, dimethylallyl-pyrophosphate; GPP, geranyl-pyrophosphate; FPP, farnesyl-pyrophosphate; GGPP, geranylgeranyl-pyrophosphate. Figure adapted from Loto et al. (2012).

Carotenoids are yellow to red pigments that are involved in photoprotective mechanisms (Alcaíno et al., 2014), among other functions. These pigments are isoprenoid compounds, which are a large and diverse family of natural products consisting of over 40,000 structurally different compounds isolated from animals, plants and microorganisms (Misawa, 2011). In non-photosynthetic eukaryotes such as yeasts, the universal isoprenoid building blocks, isopentenyl pyrophosphate (IPP, C5) and its isomer dimethylallyl pyrophosphate (DMAPP, C5), are synthesized by the mevalonate (MVA) pathway (Schmidt et al., 2011). The MVA pathway has been an important focus of study in organisms from all domains of life as many aspects of isoprenoid biosynthesis are well-conserved through evolution (Miziorko, 2011). Sterols, which are essential structural and regulatory lipids of eukaryotic cells, also derive from the MVA pathway. Emerging data suggest that undefined regulatory links exist between the carotenoid and sterol synthesis pathways. Although most genes encoding enzymes involved in the biosynthesis of carotenoids are known in *X. dendrorhous* (Figure 1), little is known about the regulation of carotenogenesis in this yeast. Several *Phaffia rhodozyma* (the anamorphic state of *X. dendrorhous*; Golubev, 1995), astaxanthin-overproducing mutants obtained by random mutagenesis showed a lower ergosterol content, the main fungal sterol, when compared to the parent strains (Miao et al., 2010, 2011).

In addition, when the *X. dendrorhous* ergosterol pathway was specifically interrupted by disrupting the *CYP61* gene, ergosterol production was blocked, and carotenoid content increased twofold (Loto et al., 2012). Even though the *cyp61*- mutants do not produce the end product ergosterol, cells accumulated other sterols and had a greater sterol content than the parental strains (Loto et al., 2012). Furthermore, *X. dendrorhous cyp61*- mutants showed increased mRNA levels for genes in the MVA pathway (Loto et al., 2012; Werner et al., 2016), suggesting that when ergosterol synthesis is inhibited, the synthesis of isoprenoid precursors increased, resulting in the overproduction of isoprenoids and isoprenoid derivatives, such as carotenoids.

Most of our current understanding of lipid homeostasis comes from mammalian cells studies where cholesterol (the analog of ergosterol in mammals), plays a fundamental role (Brown and Goldstein, 1997; Ye and DeBose-Boyd, 2011). A family of transcription factors called Sterol Regulatory Element-Binding Proteins (SREBPs) regulate sterol homeostasis. In fungal models, the SREBP pathway (Bien and Espenshade, 2010) is best studied in *Aspergillus fumigatus* (Willger et al., 2008) and the yeasts *Schizosaccharomyces pombe* (Hughes et al., 2005) and *Cryptococcus neoformans* (Chang et al., 2007; Willger et al., 2008). SREBPs are called as Sre1 in *S. pombe* and in *C. neoformans*, and as SrbA in *A. fumigatus*. SREBPs are synthesized in an inactive form as they are anchored to the endoplasmic reticulum (ER) through two transmembrane segments, where the N-terminal and the C-terminal domains face the cytoplasm. The transcription factor domain of Sre1 (the N-terminal domain, Sre1N) has a basic helix-loop-helix (bHLH) leucine zipper DNA binding motif (Hughes et al., 2005) and the C-terminal domain, the regulatory domain, interacts with a protein called SCAP (SREBP cleavage activating protein, named Scp1 in fungi) that binds sterols when the cellular sterol levels are sufficient. When sterol levels decrease, the Sre1-Scp1 complex is transported to Golgi apparatus where Sre1 is processed by proteolytic cleavage releasing Sre1N that translocates to the nucleus (Bien and Espenshade, 2010). In *S. pombe* and *C. neoformans*, Sre1N activates the transcription of genes involved in the biosynthesis of sterols, lipids and hypoxia (Bien and Espenshade, 2010). In addition, Sre1 is essential for growth in the presence of inhibitors of the synthesis of ergosterol such as azoles and compactin (Hughes et al., 2005). In *A. fumigatus*, SrbA is critical for growth under hypoxic conditions, it is involved in ergosterol biosynthesis and resistance to the azole drugs. Interestingly, *srbA* null mutants were incapable of causing disease in murine models of invasive pulmonary aspergillosis, demonstrating its role in fungal pathogenesis (Willger et al., 2008).

Considering the facts described above suggesting that sterol and carotenoid biosynthesis in *X. dendrorhous* may be regulated by a common mechanism controlled by sterols, we investigated the function of *X. dendrorhous* SREBP. Here, we present the identification and functional characterization of the *X.* *dendrorhous* *SRE1* gene. Our results demonstrate that in addition to the conserved role of Sre1 in regulation of the ergosterol biosynthesis, in *X. dendrorhous*, this protein also acts on the biosynthesis of carotenoids by directly regulating expression of mevalonate pathway genes.

## Materials and Methods

### Strains and Culture Condition

All strains used and generated in this work are listed in Table 1. The *X. dendrorhous* wild-type strain CBS 6938 (ATCC 96594) and strain CBS.*cyp61*^-^ (Loto et al., 2012), were both used as parental strains for mutant generation. For growth curves and phenotypic analysis, the parental strains and their respective mutants (CBS 6938, CBS.*sre1^-^*, CBS.*cyp61^-^*, CBS.*cyp61^-^/sre1^-^* and CBS.*gSRE1N*) were cultured in YM medium (1% glucose, 0.3% yeast extract, 0.3% malt extract, and 0.5% peptone) at 22°C with constant agitation (160 rpm) in triplicate. The optical density of the cultures at 600 nm (OD_600__nm_) was registered using a V-630, JASCO spectrophotometer. Data were analyzed with the GrowthCurver tool of R Bioconductor, which adjusts the values to a logistic function of growth (Sprouffske and Wagner, 2016). From each strain, the kinetic parameters: carrying capacity (maximum OD_600nm_, k), growth rate (μ) and generation time (Tg), were obtained. After 120 h of cultivation (stationary phase of growth), cell pellets were collected, washed with distilled water and stored at -80°C for carotenoids, sterols and RNA extraction, as well as yeast dry weight determination.

**Table 1:** Strains and plasmids used and/or constructed in this work.

	Relevant feature	Reference or source
**Strains**		
***E. coli***:		
DH5α	Strain used for molecular cloning.	Sambrook and Russell, 2001
BL21 (DE3)	Strain used for gene expression.	Novagen (Merck Millipore, Billerica, MA United States).
***X. dendrorhous***:		
CBS 6938	ATCC 96594, wild-type.	ATCC
CBS.*sre1*^-^	*sre1*^-^ mutant strain derived from CBS 6938. Resistant to zeocin.	This work
CBS.*cyp61*^-^	*cyp61*^-^ mutant strain derived from CBS 6938. It does not produce ergosterol and overproduces sterols and carotenoids. Resistant to hygromycin B.	Loto et al., 2012
CBS.*cyp61*^-^/*sre1*^-^	*sre1*^-^ mutant strain derived from CBS.*cyp61*^-^. It does not produce ergosterol. Resistant to hygromycin B and zeocin.	This work
CBS.*gSRE1N*	Derives from CBS 6938. The *SRE1* native gene, was replaced by a version that express the N-terminal domain of Sre1 (Sre1N) fused to the FLAGx3 epitope at its N-terminal. Resistant to zeocin.	This work
***S. pombe***:		
KGY425	ATCC 96115. *S. pombe* parental strain (*h- his3-D1 leu1-32 ura4-D18 ade6-M210*). Uracil auxotroph.	ATCC
Sp.sre1^-^	*sre1*^-^ mutant strain derived from KGY425 used for heterologous complementation analysis. Resistant to G418 and uracil auxotroph.	Hughes et al., 2005
Sp.*sre1*^-^ + *EV*	Strain Sp.*sre1*^-^ containing pSLF102 (*EV, empty vector*). Resistant to G418.	Hughes et al., 2005
Sp.*sre1*^-^ + *SRE1N_Sp*	Strain Sp.*sre1*^-^ containing pEP145 plasmid. Resistant to G418.	Hughes et al., 2005
Sp.*sre1*^-^ + *SRE1N_Xd*	Strain Sp.*sre1*^-^ containing pSP102_*XdSRE1N*. Resistant to G418.	This work
***S. cerevisiae***:		
S288c	Haploid reference strain used for plasmid construction by DNA assembler.	ATCC 204508
**Plasmids:**		
pBlueScript SK- (pBS)	Cloning vector (Amp*R*, ColE1 ori, white/blue colony selection).	Agilent Technologies (Santa Clara, CA, United States)
pET-TEV	Modified from pET28a (+) (Novagen, Merck Millipore, Billerica, MA United States), the thrombin cleavage site was replaced with the tobacco etch virus (TEV) cleavage site.	Fuentealba et al., 2016
pET-TEV + *XdSRE1_bHLH*	PET-TEV-derived plasmid containing 387 bp encoding the bHLH DNA binding motif of Sre1 from *X. dendrorhous* (129 aa).	This work
pMN-*hph*	pBS containing at the *Eco*RV site a hygromycin B resistance cassette (1.8 kb) to select *X. dendrorhous* transformants.	Niklitschek et al., 2008
pIR-*zeo*	pBS containing at the *Eco*RV site a zeocin resistance cassette (1.2 kb) to select *X. dendrorhous* transformants	Loto et al., 2012
pXd-*sre1*^-^::*zeo*	pBS containing at the *Eco*RV site, 1,100 bp upstream and 338 bp downstream of the *SRE1* gene and the zeocin resistance cassette between them.	This work
pXd-*gSRE1N-zeo*	Plasmid constructed by DNA assembler to express the *X. dendrorhous* FLAG-tagged Sre1N in the yeast. It contains 1,869 nucleotides (from start codon) of the *SRE1* genomic version encoding Sre1N fused to a sequence encoding the epitope FLAGx3 at the 5′ end. This sequence is flanked by the SRE1 upstream and downstream regions to direct its genome integration at the *SRE1* locus and including a zeocin resistance cassette for transformant selection.	This work
pYES2	*S. cerevisiae* expression vector containing 2 μ origin for high-copy maintenance. This plasmid was used to amplify by PCR the 2 μ DNA, which was then used for plasmid construction by DNA assembler.	Invitrogen
pFA6a	Yeast genomic targeting plasmid for gene deletion with URA3-kanMX6 double marker. This plasmid was used to amplify by PCR the kanMX6 marker, which was then used for plasmid construction by DNA assembler.	Onishi et al., 2013
pFlagTEM1	Expression plasmid containing a DNA sequence encoding a FLAG-tagged lactamase. Used to 3xFLAG epitope (3xDYKDDDDK) encoding sequence amplification through PCR for DNA assembler methodology.	Raffatellu et al., 2005


### Identification and Characterization of SRE1 Gene From *X. dendrorhous*

The *X. dendrorhous SRE1* genomic and cDNA versions were identified by translated BLAST (tblastn) analyses over genomic and transcriptomic database of strain UCD 67-385 (Baeza et al., 2015) using as query *SRE1* homologous genes from other organisms (Silver et al., 2004; Chang et al., 2007; Willger et al., 2008; Bien and Espenshade, 2010). General bioinformatic analyses were performed using the CLC Genomic Workbench v11^1^ and Geneious 10 (Kearse et al., 2012) software.

To identify sequences potentially recognizable by Sre1 in the promoter region of putative target genes, 1,000 nucleotides immediately upstream of the start codon of each candidate gene were considered. Among genes analyzed were those involved in the mevalonate pathway, carotenogenesis and ergosterol biosynthesis. For their analysis, two programs that predict transcription factor binding sites were used: TFBIND (Tsunoda and Takagi, 1999) and JASPAR (Mathelier et al., 2016). The programs TFBIND use the TRANSFAC Database (Wingender et al., 1996) and JASPAR, the JASPAR Database (Khan et al., 2018).

### Plasmid Construction and *X. dendrorhous* Transformation

Standard molecular biology methods such as PCR reactions, enzyme digestions, ligation reactions, *Escherichia coli* transformations and agarose gel electrophoresis, were performed according to the Molecular Cloning Manual (Sambrook and Russell, 2001). The *Pfu* DNA polymerase (Invitrogen, AmbionTM, Thermo Fisher Scientific Inc., Waltham, MA, United States), T4 DNA ligase and restriction endonucleases (Fermentas, Thermo Fisher Scientific Inc. Waltham, MA, United States) were used according to the manufacturers’ instructions. For plasmid DNA purification from *E. coli*, the GeneJET^TM^ Plasmid Miniprep Kit (Thermo Fisher Scientific Inc., Waltham, MA, United States) was used. Purification of DNA fragments from agarose gels was performed using the GenCatch^TM^ Advanced Gel Extraction Kit (Epoch Life Science, Inc., Missouri City, TX, United States) following the manufacturer’s recommended protocol. All plasmids and primers used in this work, are listed in Table 1 and Supplementary Table S1, respectively.

To mutate the *X. dendrorhous* *SRE1* gene by double homologous recombination (Supplementary Figure S1A), plasmid pXd-*sre1^-^*::*zeo* was constructed. First, approximately 850 bp of the up- and down-stream DNA region of the *SRE1* gene were independently PCR-amplified from strain CBS 6938 genomic DNA using the primer pairs: Sre_Del1_Out.F + 8_Int3 + HpaI.R and Sre1_Down + HpaI.F + Sre_Del2.R, respectively. Then, the up- and down-stream DNA regions were joined by overlap extension PCR (OE-PCR) (Horton et al., 1989), leaving a *Hpa*I restriction site between them according to the used primers design. The OE-PCR product was inserted at the *Eco*RV site of plasmid pBS (Sambrook and Russell, 2001) and finally a zeocin resistance cassette (mZeo^R^) (Loto et al., 2012) was inserted at the *Hpa*I site between the up- and down-stream regions of the *SRE1* gene. The transformant DNA (*SRE1* upstream region - zeocin resistance cassette - *SRE1* downstream region) was released from pXd-*sre1^-^*::*zeo* by *Xba*I and *Kpn*I digestion.

To replace the *X. dendrorhous* *SRE1* gene by its version that only encodes the N-terminal domain (Sre1N), plasmid pXd-*gSRE1N-zeo* (Supplementary Figure S1B) was constructed *in vivo* in *Saccharomyces cerevisiae* using the *DNA assembler* methodology (Kuijpers et al., 2013). First, nine PCR DNA fragments were amplified using primer pairs designed in order to allow their assembling *in vivo* through homologous recombination in *S. cerevisiae*. The PCR fragments including the primers pair and the template DNA in brackets, were: (i) the *S. cerevisiae* 2μ origin (B.Fw + B.Rv, amplified from pYES2 plasmid), (ii) the ColE1 origin and ampicillin resistance for *E. coli* (A.Fw + A.Rv, amplified from plasmid pBS), (iii) the kanMX6 resistance cassette (H.Fw + H.Rv, amplified from pFA6a), (iv) 829 bp of the upstream *SRE1* gene region (C.Fw + C_2.Rv, amplified from CBS 6938 genomic DNA), (v) 1,869 bp from the *SRE1* gene start codon [SRE1_ATG.Fw + SRE1N(stop)+term.Rv, amplified from CBS 6938 genomic DNA], (vi) 3xFLAG epitope (3xDYKDDDDK) encoding sequence (D_2.Fw + D_2.Rv, amplified from pFlagTEM1), (vii) 338 bp of the *SRE1* gene terminator region [E_Nt(stop).Fw + E.Rv, amplified from CBS 6938 genomic DNA], (viii) the zeocin resistance cassette for *X. dendrorhous* transformant selection (F.Fw + F.Rv, amplified from pIR-*zeo* plasmid) and (ix) 500 bp of the *SRE1* gene downstream region (G.Fw + G.Rv, amplified from CBS 6938 genomic DNA). Cells of *S. cerevisiae* were transformed with approximately 100 ng of each PCR product and assembled plasmids were recovered from transformants by total DNA extraction followed by *E. coli* transformation. Plasmids were recovered from *E. coli* and evaluated by PCR, restriction analyses and DNA sequencing.

*Escherichia coli* and *X. dendrorhous* were transformed by electroporation under the following conditions: 2.1 kV – 100 Ω – 25 μF, and 0.45 kV – 600 Ω – 125 mF, respectively. Electrocompetent yeast cells were prepared according to Gutiérrez et al. (2015) and transformed with 5–10 μg of DNA obtained from plasmids pXd-*sre1^-^*:*:zeo* and pXd-*gSRE1N-zeo* digested with *Not*I/*Apa*I and *Hpa*I, respectively. *X. dendrorhous* transformants were selected on YM-agar plates (1.5% agar) supplemented with 45 μg/ml zeocin. Total DNA extraction from *X. dendrorhous* was performed as previously described (Cifuentes et al., 1997).

*Saccharomyces cerevisiae* and *S. pombe* transformations and manipulations were performed using standard techniques (Sabatinos and Forsburg, 2010). Electrocompetent cells were electroporated at 1.5 kV – 200 Ω – 25 μF.

### Carotenoid and Sterol Extraction and RP-HPLC Analyses

Carotenoids and sterols were extracted from cellular pellets according to An et al. (1999) and Loto et al. (2012), respectively, and then quantified spectrophotometrically and normalized to the dry weight of the yeast. Briefly, carotenoids were quantified at 465 nm using an absorption coefficient of A_1%_ = 2,100 and sterols at 280 nm using an absorption coefficient of A_1%_ = 11,500. The extracted carotenoids and sterols were separated by RP-HPLC using a C-18 Lichrocart 125-4 column (Merck) with acetonitrile: methanol: isopropanol (85: 10: 5, v/v/v) and methanol: water (97: 3, v/v) as the mobile phase, respectively, using a 1 ml/min flux under isocratic conditions. Carotenoids and sterols were identified according to their retention time and absorption spectra compared to standards.

### RNA Extraction, Single Strand DNA Synthesis and Quantitative PCR (RT-qPCR)

Total RNA from *X. dendrorhous* was extracted according to a modified protocol from Chomczynski and Sacchi (1987). In brief, cellular pellets were broken mechanically with about 100 μL of 0.5 mm glass beads using vortex agitation during 10 min. Then, 800 μL of TriReagent (InvitrogenTM, Thermo Fisher Scientific Inc., Waltham, MA, United States) was added and mixed for 5 min in vortex agitation followed by 10 min incubation at room temperature. Chloroform, 200 μL, was added and gently mixed by inverting the tube 4–6 times, then it was incubated for 6 min at room temperature followed by a 5 min centrifugation at 4,000 × g. The aqueous phase was recovered, transferred to a clean tube and RNA was precipitated with 1 volume of isopropanol 80% for 15 min at room temperature. Then, RNA was washed twice with 70% ethanol, suspended in 30–60 μL of Milli-Q H_2_O and quantified at 260 nm in a Take3 microplate for Epoch 2 spectrophotometer (BioTek Instruments, Winooski, VT, United States).

The single strand DNA (cDNA) synthesis was carried out according to the M-MLV reverse transcriptase (InvitrogenTM, Thermo Fisher Scientific Inc., Waltham, MA, United States) manufacturer’s recommended protocol, using 5 μg of total RNA, 1.25 μM oligo-dT18 primer, 0.5 μM dNTPs and 200 U of M-MLV reverse transcriptase in a final volume of 20 μl. The relative transcript level determinations were done in an Mx3000P quantitative PCR system (Stratagene, Applied Biosystems, Foster, CA, United States) using specific primers pairs (Supplementary Table S1) having efficiencies greater than 95%, as determined by standard curves with a correlation coefficient of *R*^2^ ≥ 0.996. Each reaction contained 1 μL of the reverse transcription reaction, 0.25 μM of each primer and 10 μL of the SsoFast EvaGreen Supermix kit (Bio-Rad, Hercules, CA, United States) in a final volume of 20 μL. The obtained Ct values were normalized to the respective value of the β-actin gene [GenBank: X89898.1] (Wery et al., 1996) and later expressed as a function of the control conditions (wild-type strain) using the ΔΔCt algorithm (Livak and Schmittgen, 2001).

### Electromobility Shift Assay (EMSA)

EMSAs were performed using the deduced DNA-binding bHLH motif protein of the *X. dendrorhous* Sre1 (residues 349 to 477) fused to a His-tag, which was synthesized in *E. coli* BL21 (DE3) using plasmid pET-TEV (Fuentealba et al., 2016). Gene expression was induced with 1 mM IPTG using exponential growth cultures grown in LB medium at 22°C containing the corresponding antibiotics. Cells were collected, washed and suspended in buffer A (20 mM Tris, 0.5 M NaCl, 20% glycerol, 5 mM Imidazole, pH: 7.4) and broken using a Branson sfx 550 ultrasonic homogenizer (EmersonTM, St. Louis, MO, United States). The homogenate was centrifuged at 18,407 ×*g* for 10 min at room temperature and analyzed by SDS-PAGE to confirm the presence of the recombinant protein in relation to the non-induced control. The protein was purified under non-denaturing conditions using NI-Sepharose affinity chromatography (HisTRap FF crude, GE Healthcare Bio-Sciences, Pittsburgh, PA, United States) and the purified protein was eluted from the column using buffer A containing 0.25 M imidazole and collecting fractions of 1 ml. Protein quantification was carried out using the BCA Protein Assay Kit (PierceTM, Thermo Fisher Scientific Inc., Waltham, MA, United States).

For DNA-protein binding reactions, 60 bp DNA probes labeled with biotin at their 3′ ends were used including two potential SRE sequences deduced by bioinformatics analysis of the *HMGS* gene [GenBank: MK368600] promoter region. Probes were constructed by joining two fragments of 30 bp upstream the *HMGS* gene translation initiation codon (bases -597 to -568 and bases -238 to -209), each one containing a potential SRE sequence. A total of four probes were used, each one containing a different combination of wild-type or mutated SREs, which were made by annealing two fully complementary oligonucleotides in the following buffer 10 mM Tris-HCl, 1 mM EDTA, 50 mM NaCl, pH 8.0; under the following conditions: denaturation at 95°C for 2 min, 25°C for 45 min and kept at 4°C until use. Reactions were performed using binding buffer (50 mM Hepes-KOH pH:7.5, 20% v/v glycerol, 100 mM KCl, 5 mM MgCl_2_, 1 mM DTT, 0.8 mg/ml BSA, 0.3 mg/ml poly(dI-dC) containing 1.7 nM of the recombinant protein and 0.5 nM of DNA probe in a final volume of 10 μl. First, reactions containing the poly (dIdC) and the recombinant protein were incubated at room temperature for 30 min, then the DNA probe and competitor DNA (unlabeled probe) were included and incubated at 22°C for 30 min. DNA-protein complexes were resolved by 10% native polyacrylamide gel electrophoresis in TBE buffer 0.5X, run at 75 V for 2.5 h at 4°C and transferred to a nylon membrane IMMOBILON-NY+ (Millipore, Burlington, MA, United States) with a Trans-Blot Semi-Dry (BIORAD, Hercules, CA, United States) under the following conditions: 5 V for 5 min, 10 V for 10 min, 15 V for 10 min and 20 V for 5 min. DNA was immobilized by UV (312 nm) exposition for 15 min and the labeled DNA was visualized with the LightShiftTM Chemiluminescent EMSA Kit (Thermo Scientific, Thermo Fisher Scientific Inc., Waltham, MA, United States) using a Imaging platform ALLIANCE 9.7 (UVITEC Cambridge, Cambridge, United Kingdom).

### Chromatin Immunoprecipitation (ChIP) – PCR Assay

For ChIP assays, strains CBS.g*SRE1N* and CBS.*sre1^-^* were grown until late exponential phase and 10 ml culture samples were collected in 50 ml conical centrifuge tubes to be crosslinked with formaldehyde (37%) (Merck KGaA, Darmstadt, Germany) to a final concentration of 1%, and then incubated for 30 min in a tube rotator at room temperature. Crosslinking was stopped by adding glycine at 0.15 mM (final concentration) for 15 min. Then, mixtures were centrifuged, and pellets washed twice with cold phosphate buffered saline (2.56 g Na_2_HPO_4_ ⋅ 7H_2_O, 8 g NaCl, 0.2 g KCl and 0.2 g KH_2_PO_4_, pH 7.4), aliquoted in 2 ml screw cap tubes and stored at -80°C until use or treated as described below. To each pellet, 0.5 mm glass beads and ChIP lysis buffer sc-45000 (Santa Cruz Biotechnology Inc, Dallas, TX, United States) were added and subjected to mechanical cell rupture using a Mini-beadbeater-16 (BioSpec Products Inc., Bartlesville, OK, United States) for 30 s followed by ice incubation for 2 min (7 cycles in total). Cell debris was gently rescued with a micropipette and centrifuged at 4°C for 5 min at 600 ×*g*. Then, the pellet was resuspended in ChIP lysis buffer high salt sc-45001 (Santa Cruz Biotechnology Inc, Dallas, TX, United States), sonicated for 10 s followed by 2 min incubation in ice (6 cycles in total) using Branson sfx 550 ultrasonic homogenizer (EmersonTM, St. Louis, MO, United States) and centrifuged at 4°C for 10 min at 9,300 ×*g*. The supernatant (chromatin) was rescued and divided in three aliquots of 200 μL each; chromatin in each aliquot was precleared with 50 μl of protein A/G PLUS-agarose sc-2003 (Santa Cruz Biotechnology Inc, Dallas, TX, United States) for 1.5 h incubation at 4°C in a tube rotator. Then, the pre-clarified chromatin was rescued by centrifugation and the three aliquots were treated as follow: (i) stored at -20°C (input DNA), (ii) incubated with 5 μg of the monoclonal antibody ANTI-FLAG^®^ M2 (catalog number F1804; Merck KGaA, Darmstadt, Germany) and (iii) used as control without antibody. The two last samples were incubated at 4°C overnight and then, 50 μl of protein A/G PLUS-agarose sc-2003 (Santa Cruz Biotechnology Inc, Dallas, TX, United States) was added and incubated at 4°C for 2 h. Beads were harvested by centrifugation at 13,500 ×*g* for 20 s and washed four times with ChIP lysis buffer high salt sc-45001 and ChIP wash buffer sc-45002 (Santa Cruz Biotechnology Inc, Dallas, TX, United States). After the final wash, beads were transferred to a new tube, resuspended in elution buffer sc-45003 (Santa Cruz Biotechnology Inc, Dallas, TX, United States) and incubated at 55°C for 2 h. Chromatin was recovered and incubated at 65°C overnight to reverse the crosslink (the input DNA sample was treated in the same way). Finally, all DNA samples was purified by phenol: chloroform: isoamyl alcohol (25: 24: 1) and chloroform: isoamyl alcohol (24: 1) extractions using standard technique (Sambrook and Russell, 2001).

For PCR amplification, primers flanking the two potential *SRE* sequences at the promoter region of the *HMGS* gene (located at -584 and -232 bp upstream of the translation start codon) and four potential *SRE* sequences at the promoter region of the *HMGR* gene [GenBank: MK368599] (located at -775, -770, -769, and -753 bp upstream of the translation start codon), were designed (Supplementary Table S1). As a control, the promoter region of *X. dendrorhous* *grg2* gene [GenBank: JN043364.1] was used as, to the best of our knowledge, there are no reports of this gene being regulated by SREBP/Sre1 in other organisms and no potential SRE sequences were predicted at its promoter through bioinformatics analysis.

### RNA-seq and Bioinformatics Analyses

RNA-seq analysis of the CBS 6938 (wild-type) and CBS.*sre1^-^* strains cultured in YM medium for 40 h at 22°C and then, cells were harvested to extract total RNA. RNA integrity was evaluated using a Bioanalyzer 2100 (Agilent, Madrid, Spain), 75 bp libraries were constructed and paired-end sequenced using the Illumina HiSeq 2000 platform (Illumina, San Diego, CA, United States) at the National Center for Genomic Analysis of Barcelona, Spain (CNAG). After quality controls, sequence reads were mapped to 6,385 protein coding genes (Sharma et al., 2015) using the RNA-seq analysis tool from the CLC Genomics Workbench v11 program to determine count data and transcripts per million (TPM) values. Differentially expressed Genes (DEGs) were estimated using the DESeq2 package of R Bioconductor (Love et al., 2014) using the criteria of an adjusted *p*-value < 0.05.

## Results

### Identification and Molecular Characterization of the *X. dendrorhous* SRE1 Gene

**FIGURE 2:**
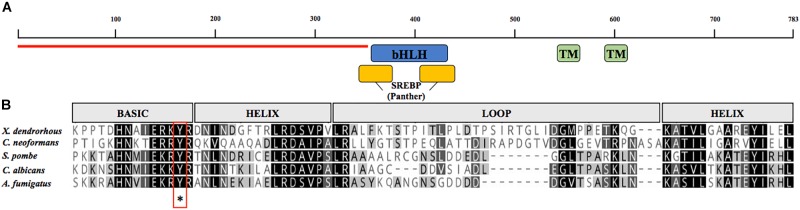
Analysis of the amino acid sequence encoded by the *SRE1* gene of *X. dendrorhous*. **(A)** Representation of the predicted conserved domains: two transmembrane domains (green; TMHMM database), the DNA binding domain type bHLH (blue; SMART database), sterol regulatory element-binding protein motif domain (yellow; PTHR12565, Panther database) and N-terminal domain rich in serine and proline residues (indicated with red line). **(B)** Alignment of bHLH domain of Sre1 from different fungi using Geneious Alignment tools with matrix Blossum62. The five sequences contain a conserved tyrosine residue (indicated by asterisk) specific to the SREBP family of bHLH transcription factors.

Sequence searches for a potential *SRE1* gene using *X. dendrorhous* genome and transcriptomic databases (Baeza et al., 2015) were performed using as queries the sequences of *S. pombe* [NP_595694,], *C. neoformans* [XP_567526], and *A. fumigatus* [XP_749262]. Using this approach, a candidate *SRE1* gene was identified [GenBank: MK368598], which consists of 2,816 bp (from the start to the stop codon) composed by 4 exons giving an ORF of 2,352 bp and a predicted Sre1 protein of 783 amino acids. The deduced protein contains a bHLH DNA binding motif in its N-terminal domain (residues 356–449). Notably, the Sre1 bHLH domain contains a conserved tyrosine residue, which distinguishes SREBPs from other bHLH transcription factors (Párraga et al., 1998). In addition, the N-terminal region of Sre1 that precedes the bHLH motif is rich in serine and proline residues, compromising 35% of the total amino acids of the N-terminal domain. Secondary structure predictions and hydrophobicity profiles of Sre1 revealed two possible transmembrane helixes between residues 525–547 (TM1) and 588–613 (TM2) (Figure 2). A domain of unknown function (DUF2014) that may be important for interaction with SCAP is present in several fungal SREBPs (Maguire et al., 2014). The DUF2014 domain was not found in the *X. dendrorhous* Sre1 protein, similar to Sre1 proteins from other basidiomycetes such as *C. neoformans* (Maguire et al., 2014). In summary, the predicted Sre1 protein from *X. dendrorhous* has the characteristic features of SREBP/Sre1 proteins described in other organisms.

**FIGURE 3:**
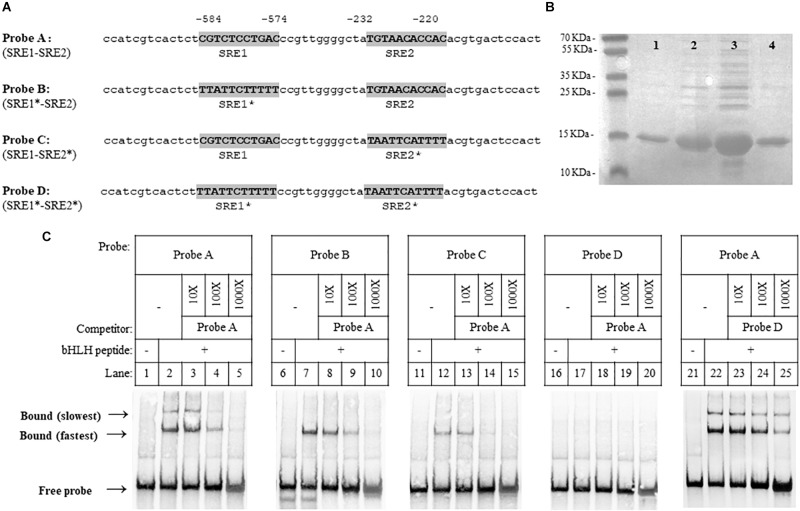
*Xanthophyllomyces dendrorhous* Sre1 binding to DNA. Sre1 binding to DNA was evaluated by employing DNA fragments of 60 bp in length, which were designed from the *X. dendrorhous* *HMGS* gene promoter containing 2 predicted SRE elements. **(A)** Probe sequences are shown: wild-type (probe A) and mutants (probes B to D). The two potential SRE elements (SRE1 and SRE2) are highlighted in gray and the (^∗^) after SRE1 or SRE2 indicates if the corresponding SRE element is mutated. Numeration above the sequences is relative to the *HMGS* gene translation initiation ATG codon. **(B)** SDS-polyacrylamide gel electrophoresis of different fractions of purified recombinant Sre1 protein (residues 349 – 477) containing the bHLH DNA-binding motif of *X. dendrorhous* Sre1, 4 fractions are shown as an example. Gel was stained with Coomassie blue and fraction 3 (lane 3) was used in the EMSA assays. **(C)** EMSA assays. Purified recombinant bHLH DNA-binding Sre1 protein was mixed with the indicated biotinylated DNA probes: probe A (lanes 2–5 and 22–25), probe B (lanes 7–10), probe C (lanes 12–15) and probe D (lanes 17–20), including the corresponding controls without the bHLH protein (lanes 1, 6, 11, 16, and 21). Assays were performed in the absence (lanes 2, 7, 12, 17, and 22) or presence (lanes 3–5, 8–10, 13–15, 18–20, and 23–25) of unlabeled competitor DNA probes. Competitor probes were used at a 10-fold excess (10 X, lanes 3, 8, 13, 18, and 23), a 100-fold excess (100X, lanes 4, 9, 14, 19, and 24), and a 1,000-fold excess (100X, lanes 5, 10, 15, 20, and 25). As competitors, probes A (lanes 3–5, 8–10, 13–15, and 18–20) and D (lanes 23–25), were used.

In our previous studies, potential SRE sequences, which are conserved SREBP DNA binding sequences, were identified in the promoter regions of *X. dendrorhous* genes of the MVA pathway (Werner et al., 2016) and genes encoding P450s enzymes (Córdova et al., 2017). Considering that *HMGS* (hydroxymethylglutaryl-CoA synthase) of the MVA pathway is a well-known target of SREBPs in organisms like *S. pombe* (Hughes et al., 2005), *C. neoformans* (Chang et al., 2007) and mammals (Smith et al., 1988; Goldstein and Brown, 1990; Wang et al., 1994; Horton et al., 2003), we analyzed the promoter region of this gene and identified two potential SRE sequences (SRE1: CGTCTCCTGAC and SRE2: TGTAACACCAC, located at positions -584 to -574 and -232 to -220 from the translation start codon, respectively). To test whether the *X. dendrorhous* Sre1 gene product binds directly to these sequences, we performed electromobility shift assays using 60 bp biotin-labeled DNA probes designed from the *HMGS* gene promoter containing different combinations of wild-type or mutated SREs (Figure 3A). For this, the DNA binding domain of the *X. dendrorhous* Sre1 protein (residues 349–477), was expressed and purified from *E. coli* (Figure 3B).

The DNA probe containing both predicted SRE sequences showed electrophoretic mobility shift when incubated with the purified protein (Figure 3C, lane 2), and two shift bands were observed. The electrophoretic mobility shift of both bands was reduced when the protein was incubated with increasing concentrations of the non-labeled wild-type competitor probe. To confirm whether Sre1 bound to both SREs, we tested its binding to probes with either (probes B and C) or both (probe D) of the SRE sequences previously mutated (Figure 3C, lanes 6–15). When using probes with one SRE sequence mutated, a single shift band (coinciding with the fastest migrating band detected when using the probe with both wild-type SREs), was observed. No electrophoretic mobility shift was observed using the probe with both mutated SREs. These results suggest that the Sre1 protein binds to both SRE sequences resulting in three different DNA-protein complexes: the Sre1 protein bound to both SREs (the slowest migrating band) and bound to SRE1 or to SRE2 (the fastest migrating band). Probably, Sre1 binding to SRE2 is stronger than to SRE1, because a higher concentration of the competitor probe was required to block its binding. Finally, increasing concentrations of the non-labeled competitor probe with both mutated SREs did not block protein binding to the probe with both wild-type SREs (Figure 3C, lanes 21–25). These results indicate that the DNA binding domain of the *X. dendrorhous* Sre1 binds directly and specifically to these two SRE sequences from the *HMGS* promoter *in vitro*.

### *Xanthophyllomyces dendrorhous* SRE1 Gene Functionality Studies

**FIGURE 4:**
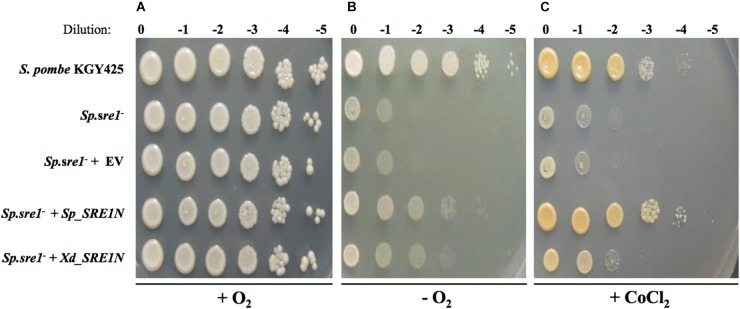
Growth phenotype of *S. pombe* strains. Serial dilutions of cultures of *S. pombe* strains KGY425 (parental)*, Sp.sre1^-^, Sp.sre1^-^ +* EV (empty vector)*, Sp.sre1^-^ + Sp_SRE1N* (vector expressing Sre1N from *S. pombe*) and *Sp.sre1^-^ + Xd_SRE1N* (vector expressing Sre1N from *X. dendrorhous*), were seeded on YES medium and incubated at 30°C under **(A)** normoxia for 3 days, **(B)** hypoxia using the AnaeroGen de Oxoid^®^ anaerobiosis system for 10 days and **(C)** YES medium supplemented with cobalt chloride (1.6 mM) for 3 days. Dilution indicates no initial culture dilution (0) and dilutions 10^-1^ to 10^-5^ (–1 to –5).

Previously, it was demonstrated that a *S. pombe* *sre1^-^* mutant was unable to grow under anaerobic conditions (Hughes et al., 2005), or in the presence of cobalt chloride (Lee et al., 2007), an agent that mimics hypoxia conditions. However, growth under those conditions was restored when the mutant was complemented with the native gene. Given that binding to Scp1 is required for *S. pombe* Sre1 activation and that *X. dendrorhous* Sre1 lacks an identifiable DUF2014 domain, we chose to test whether the N-terminal transcription factor domain (Sre1N) could activate gene expression and rescue SREBP function in fission yeast as a first approximation to study its functionality. Serial dilutions of cultures of five *S. pombe* strains (initial titer: 5 × 10^7^ CFU/ml, approximately), were seeded on rich YES medium and incubated under normoxia, hypoxia or in the presence of cobalt chloride (Figure 4). All strains grew well and similar under normoxia conditions. As expected, growth of the *sre1^-^* mutant (strain *Sp.sre1^-^*) was affected under hypoxia and in the presence of cobalt chloride when compared to the parental strain. Growth was not restored when the mutant was transformed with the empty vector (strain *Sp.sre1^-^* + EV) unlike when it was transformed with the vector carrying the *S. pombe SRE1* gene fragment that encodes the N-terminal domain (residues 1–440) of the Sre1 protein (strain *Sp.sre1^-^* + *Sp_SRE1N*). Interestingly, an intermediate growth phenotype under hypoxia and in the presence of cobalt chloride was observed in the strain carrying the vector that expresses the N-terminal domain (residues 1–483) of the *X. dendrorhous* *SRE1* gene (strain *Sp.sre1^-^* + *Xd_SRE1N*), suggesting that the *X. dendrorhous SRE1* gene fragment that encodes Sre1N, partially complements the *sre1^-^* mutation in *S. pombe*.

Next, to study the functionality of the *SRE1* gene in *X. dendrorhous* and its potential role in the biosynthesis of carotenoids and sterols, *sre1^-^* mutants and a mutant that expresses only the N-terminal domain of the *X. dendrorhous* Sre1 protein (Sre1N, residues 1 to 524; strain CBS.*gSRE1N*), were constructed. Deletion mutants were obtained from the wild-type strain CBS 6938 (strain CBS.*sre1^-^*) and from strain CBS.*cyp61^-^* (strain CBS.*cyp61^-^*/*sre1^-^*). CBS.*cyp61^-^* is also derived from CBS 6938 and does not produce ergosterol due to the *CYP61* gene mutation, but it overproduces sterols and carotenoids (Loto et al., 2012). Thus, CBS.*cyp61^-^* is a good model in which to study the effect of *SRE1* on the biosynthesis regulation of these two types of metabolites. The designed gene replacements through homologous recombination and the presence of the corresponding resistance cassette for transformant selection, were confirmed by PCR analysis using comprehensive sets of primers (Supplementary Figure S2).

**FIGURE 5:**
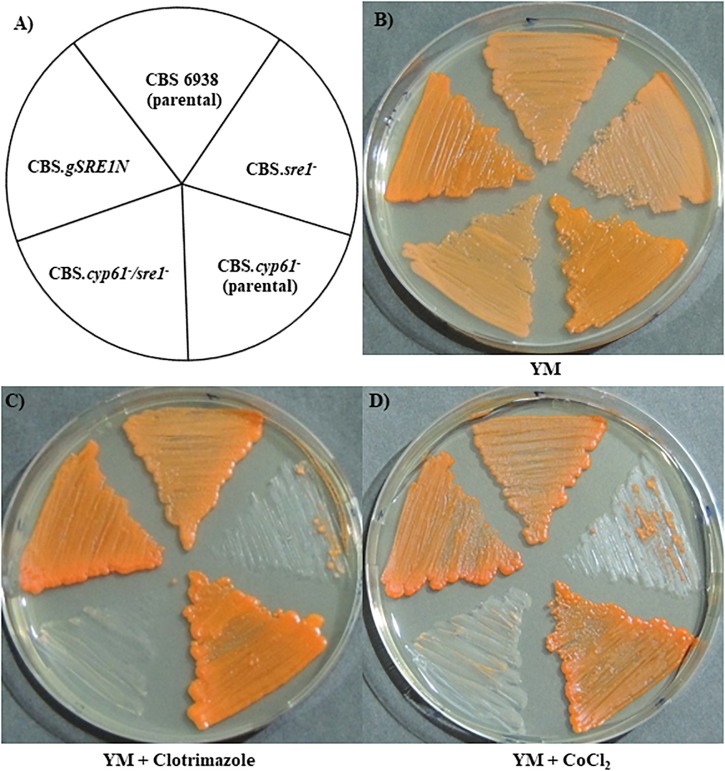
Growth phenotype of *X. dendrorhous* strains. Plates were incubated for 5 days at 22°C. **(A)** Scheme indicating the position of the seeded strains, and cultures on **(B)** YM medium and on YM medium supplemented with **(C)** clotrimazole (0.15 μg/ml) or **(D)** cobalt chloride (400 μM).

First, the five *X. dendrorhous* strains studied in this work: CBS 6938 (wild-type), CBS.*sre1^-^*, CBS.*cyp61^-^*, CBS.*cyp61^-^*/*sre1^-^* and CBS.*gSRE1N*, were seeded on YM rich medium and assayed for color phenotypes (Figure 5A,B). To the naked eye, no color difference was appreciated between strains CBS.*sre1^-^* and the wild-type. However, the same *sre1^-^* mutation in strain CBS.*cyp61^-^*/*sre1^-^* produced a lower pigmentation than its parental strain CBS.*cyp61^-^*. Interestingly, strain CBS.*gSRE1N* showed an intense pigmentation similar to that of CBS.*cyp61^-^*, indicating that expressing only the N-terminal domain of Sre1 in *X. dendrorhous* produces the same color phenotype as when mutating the *CYP61* gene. Together, these observations suggest that the SREBP pathway is activated in the *cyp61^-^* mutant. Growth in the presence of clotrimazole or chloride cobalt was also evaluated. Unlike wild-type and CBS.*cyp61^-^*, the two *sre1^-^* mutants did not grow when clotrimazole or chloride cobalt were added to the culture media (Figure 5C,D), suggesting that ergosterol biosynthesis is affected in the *sre1^-^* mutants. Strain CBS.*gSRE1N* grew similar to the wild-type strain under these conditions, demonstrating that the Sre1N is functional.

To evaluate carotenoid and sterol production, the five *X. dendrorhous* strains were cultured in YM media until the stationary phase (Supplementary Figure S3). In general, growth parameters were not affected between each mutant and the corresponding parental strain. However, strains CBS.*cyp61^-^* and CBS.*cyp61^-^/sre1^-^* showed higher growth rate (μ) and a lower generation time (Tg) than the other analyzed strains. After 120 h of culture, samples were taken to extract sterols, carotenoids and RNA.

Regarding metabolite production, both *sre1^-^* mutant strains showed a lower sterol production than their corresponding parental strains and strain CBS.*gSRE1N*, showed a higher sterol production than the wild-type (Table 2). Even though the CBS.*cyp61^-^* strain does not produce ergosterol (Loto et al., 2012), the production of total sterols was greater than in the wild-type strain (and similar to strain CBS.*gSRE1N*). However, this sterol overproducing phenotype is suppressed by the *sre1^-^* mutation. Sterol composition was evaluated by RP-HPLC, but no differences were observed between mutants and their parental strains: wild-type, CBS.*sre1^-^* and CBS.*gSRE1N* strains accumulate a single sterol (98%, approximately), which was represented by a peak around 10.5 min in the chromatogram that corresponds to ergosterol as confirmed by co-injecting the sample with commercial ergosterol. As expected, strains CBS.*cyp61^-^* and CBS.*cyp61^-^/sre1^-^* did not produce ergosterol and accumulated two others metabolites with retention times of 9 and 13 min (Table 2) that had a sterol spectrum. A similar pattern was observed in carotenoid production: in general, the production of carotenoids was reduced in the *sre1^-^* mutants in relation to their parental strains and it was higher in strain CBS.*gSRE1N* compared to the wild-type (Table 2). The higher production of carotenoids observed in strain CBS.*cyp61^-^* compared to the wild-type (about twofold higher), was reduced to wild-type levels by the *sre1^-^* mutation and strain CBS.*gSRE1N*, produced similar elevated carotenoids levels as strain CBS.*cyp61^-^.* These results are in accordance to the strains visual observations (Figure 5B). Carotenoid composition was also evaluated by RP-HPLC. In general, no significant differences were detected between the *sre1^-^* mutants in relation to their parental strains. Nevertheless, the astaxanthin fraction was higher in strain CBS.*cyp61^-^* than in the wild-type (86% versus 73%, respectively). Astaxanthin (the final product of carotenogenesis in *X. dendrorhous*) is produced from β-carotene through the production of several oxygenated intermediary carotenoids such as echinenone, hydroxy echinenone and phoenicoxanthin (Alcaíno et al., 2016) and the proportion of these intermediary carotenoids, decreased in strain CBS.*cyp61^-^* compared to the wild-type. Interestingly, carotenoid composition in strain CBS.*cyp61^-^/sre1^-^* was more similar to that of the wild-type strain than the CBS.*cyp61^-^*. Finally, strain CBS.*gSRE1N* showed a slightly lower proportion of astaxanthin (67%) and a higher proportion of β-carotene (5%) in relation to the other strains.

**Table 2:** Sterol and carotenoid production in *X. dendrorhous* strains studied in this work.

	Strain				
Metabolite	CBS 6938 (wild-type)	CBS.*sre1*^-^	CBS.*cyp61*^-^	CBS.*cyp61*^-^/*sre1*^-^	CBS.*gSRE1N*
Total sterols (mg/g; 100%)	5.6 ± 0.3^a^	4.3 ± 0.3^b^	6.2 ± 0.3^c^	4.1 ± 0.3^b^	7.0 ± 0.1^d^
% ergosterol, 10.5 min	99	97	ND	ND	95
% peak 9.0 min	ND	ND	26 ± 4	20 ± 3	ND
% peak 13.0 min	ND	ND	75 ± 5	79 ± 4	ND
Total carotenoids (μg/g, 100%)	302 ± 13^a^	269 ± 24^a^	619 ± 35^b^	304 ± 5^a^	691 ± 19^b^
% other carotenoids	12	14	12	15	15
% β-carotene	0.4	ND	ND	ND	5
% intermediary carotenoids	15	19	2	20	13
% astaxanthin	73	67	86	65	67


*Total sterols and carotenoids were normalized to the yeast dry weight in g. Peak 9.0 min and peak 13.0 min, correspond to sterol peaks observed in chromatograms and their approximate retention time. Other carotenoids refer to carotenoids such as cantaxanthin, OH-ketotorulene, gamma-carotene and others unidentified; intermediary carotenoids to carotenoids produced between β-carotene and astaxanthin in carotenogenesis. ND, not detected. Table shows the mean values of three independent cultures ± standard deviations. One-way ANOVA and Tukey *post hoc* test was used to compare the strains metabolite production; superscript letters a, b, c and d correspond to the statistical comparisons: same letter indicates no statistically significant differences and different letters indicate significant differences among strains with *p* < 0.05.*

### Sre1-Dependent Gene Expression Global Overview

To obtain a global overview of the regulatory effect of Sre1 in *X. dendrorhous*, we conducted transcriptome sequencing (RNA-seq) comparing expression profiles of the wild-type and CBS.*sre1^-^* strains to determine global changes in gene expression levels under standard culture conditions. The determination of differentially expressed genes (DEGs) between the CBS.*sre1^-^* and wild-type strains by means of DESeq2 (fold change set at > 1.5 and *p* adjusted ≤ 0.5) revealed minimal transcriptional differences with only 15 genes significantly changed. Of these 15 genes, 11 genes decreased their expression in the CBS.*sre1^-^* mutant strain and therefore could be Sre1-dependent, these genes correspond mainly to genes of the mevalonate and sterol biosynthesis pathways, such as *HMGS*, *HMGR* (3-hydroxy-3-methylglutaryl-coenzyme A reductase), *ERG6*, *ERG7*, *ERG24*, *ERG25*, and *CYP51* (Table 3).

**Table 3:** Differentially expressed genes (DEGs) between strains CBS.*sre1^-^* and the wild-type (CBS 6938) using the DESeq2 tool, adjusted *p*-values ≤ 0.05.

Accession No	Uniprot description	Gene name	Log_2_ FC CBS.*sre1^-^/* CBS 6938
**MVA Pathway:**			
CED83016	Hydroxymethylglutaryl-CoA synthase	*ERG13/HMGS*	–1.5
CED85502	Hydroxymethylglutaryl-CoA reductase	*HMGR*	–1.0
**Ergosterol Biosynthesis:**			
CED85039	C-24 methyl transferase	*ERG6*	–1.9
CED82918	Lanosterol synthase	*ERG7*	–1.3
CED84182	Lanosterol demethylase	*ERG11/CYP51*	–1.1
CED85474	C-14 reductase	*ERG24*	–1.8
CDZ96809	C-4 methyl sterol oxidase	*ERG25a*	–1.7
CED83720	C-4 methyl sterol oxidase	*ERG25b*	–1.6
**SREBP Pathway:**			
CDZ98428	Myc-type, basic helix-loop-helix (bHLH) domain	*SRE1*	–2.0
**Other genes:**			
CED83744	Hypothetical protein	NA	–1.2
CED85636	Methyl methanesulphonate-sensitivity protein 22	NA	–0.8
CDZ98429	Orotate phosphoribosyltransferase	NA	1.2
CDZ97703	Retrotransposon ty1-copia subclass	NA	1.5
CDZ96158	Gag-pol polyprotein	NA	1.5
CED84234	hypothetical protein	NA	1.7


*NA, not available.*

**FIGURE 6:**
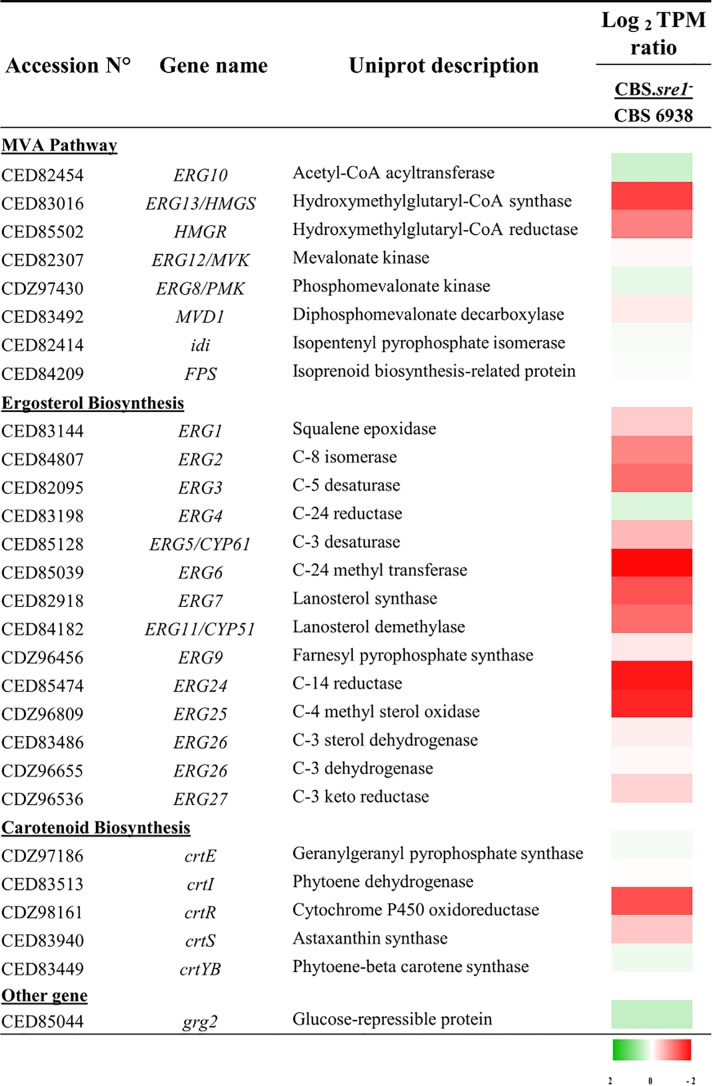
Heat map presentation of gene expression of genes of the mevalonate pathway, and sterol and carotenoid biosynthesis. The values are normalized to the wild-type strain and expressed in Log_2_ TPM (Transcripts per million). The color gradient code indicates in green, the transcripts that increase and in red, decrease.

To obtain an overview of genes of the mevalonate, carotenoid and sterol biosynthesis pathways, we analyzed the RNA-seq data by means of TPMs ratio between each culture condition (Figure 6). As observed through the DESeq2 analysis, the expression of the *HMGS* and *HMGR* genes of the mevalonate pathway and of most genes of the sterol biosynthesis decreased in the *sre1^-^* deletion when compared to the wild-type under standard culture conditions (CBS.*sre1^-^*/CBS 6938 column, Figure 6). Interestingly, the expression of gene *crtR*, which is involved in both, carotenoid and sterol biosynthesis, also decreased in the *sre1^-^* mutant.

The promoter region (1.0 kb upstream of the putative start codon) of genes involved in the mevalonate pathway, ergosterol and carotenoids biosynthesis was analyzed with the TFBIND and JASPAR tools. Potential SRE sequences were identified in several of these genes, most of them coinciding with those that showed differential expression in the RNA-seq analyses (Supplementary Table S2). Together, these perspective results, point that genes of the mevalonate pathway and of the ergosterol biosynthesis, are transcription targets of Sre1 in *X. dendrorhous*, similar as occurs in other organisms.

### The *X. dendrorhous* Sre1N Binds to Promoters of MVA Pathway Genes *in vivo*

**FIGURE 7:**
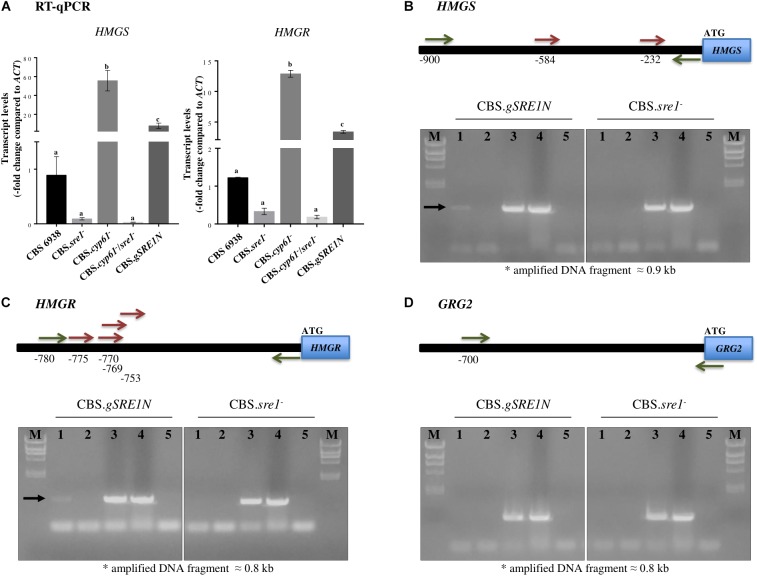
*HMGS* and *HMGR* relative transcript levels, and *X. dendrorhous* Sre1 DNA-binding *in vivo*. **(A)** Relative expression of genes *HMGS* [GenBank: MK368600] and *HMGR* [GenBank: MK368599]. The transcript levels were evaluated by RT-qPCR after 120 h of culture in YM rich medium with constant agitation and normalized to the housekeeping β-actin gene [GenBank: X89898.1]. Values are the mean ± standard deviation of three independent cultures. (One-way ANOVA and Tukey *post hoc* test; different letters indicate significant differences among strains with *p* < 0.01). ChIP-PCR assays of the **(B)** *HMGS*, **(C)** *HMGR* and **(D)** *grg2* genes from *X. dendrorhous*. Top: Representation of the amplified region of the promoter (horizontal black bar) of each gene. Predicted Sre1 binding sites (SRE: 5′-BPyCACNCCAPy-3′; B: any base except thymine, Py: pyrimidine and N: any base) are represented by red arrows; two SREs were predicted at the *HMGS* promoter (SRE 1: –584/CGTCTCCTGAC/–574 and SRE2: –232/TGTAACACCAC/–222), four at the *HMGR* promoter (SRE1: –775/TGTGACCTCAC/–765, SRE2: –770/CCTCACGTGAC/–760, SRE3: –769/CTCACGTGACTA/–758 and SRE4: –753/CGACACGTGTG/–743) and none at the *grg2* promoter. Primers used in the PCR analyses are represented by green arrows. Bottom: PCR products after ChIP of strains CBS.g*SRE1N* (mutant expressing the *SRE1* (residues 1–524) gene fragment encoding the N-terminal domain of Sre1 fused to 3 copies of the FLAG -3xDYKDDDDK- epitope encoding sequence) and CBS.*sre1^-^* (deletion mutant of SRE1 used as a negative control strain). PCR reactions were performed: lanes: (1) ChIP assay (positive results are indicated by a black arrow), (2) ChIP assay without including the antibody, (3) input DNA, (4) genomic DNA from the wild-type yeast strain CBS 6938, (5) without DNA. M: Lambda *Hind*III marker (23.2, 9.4, 6.6, 4.4, 2.3, 2.0, and 0.6 kb).

Considering that the *HMGS* and *HMGR* of the mevalonate pathway are well known targets of SREBPs/Sre1 in different organisms, their expression at the mRNA level was further studied by RT-qPCR in the five yeast strains studied in this work. Transcript levels of both genes were highly induced in strains CBS.*cyp61^-^* and CBS.*gSRE1N* compared to the wild-type, whereas mutation of *SRE1* blocked induction in double mutant CBS.*cyp61^-^*/*sre1^-^* (Figure 7A).

We also tested if *HMGS* and *HMGR* genes were direct transcription targets of Sre1N. Gene *grg2* was selected as a negative control as there are no evidence supporting that this gene could be a SREBP/Sre1 target. Moreover, it should be noted that the *grg2* gene expression, did not significantly change in the *sre1^-^* mutant compared to the wild-type (Figure 6), supporting to be a good negative control. For this, ChIP-PCR assays were performed using strain CBS.*gSRE1N* as this was designed in such way to produce the Sre1N protein fused to three copies of the epitope FLAG (3xDYKDDDDK) at its N-terminal. Strain CBS.*sre1^-^* was used as a negative control. As expected, the promoter regions of the *HMGS* (with two predicted SREs) and *HMGR* (with four predicted SREs) genes were PCR-amplified only when using immunoprecipitated DNA of strain CBS.g*SRE1N*, but not when using the negative control strain (Figure 7B,C, lanes 1), indicating that Sre1N binds *in vivo* to the promoter region of these genes. No amplification product was obtained for the promoter region of gene *grg2* when using immunoprecipitated DNA from strain CBS.g*SRE1N* or from the negative control strain (Figure 7D, lanes 1). Together, these results indicate that genes *HMGS* and *HMGR* of the mevalonate pathway are direct targets of Sre1N in *X. dendrorhous*.

## Discussion

Little is known about the transcriptional regulation of carotenoid biogenesis. Recent work from our laboratory suggested that sterol levels participate in this process in *X. dendrorhous* at the level of the synthesis of carotenoid precursors (Loto et al., 2012; Leiva et al., 2015; Werner et al., 2016). Carotenoids and sterols derive from the mevalonate pathway suggesting that they could share regulatory mechanisms. SREBPs transcriptional factors are conserved regulators of sterol homeostasis that control expression of mevalonate pathway genes in fungi (Bien and Espenshade, 2010), and we thus considered SREBP as a strong candidate for acting in the regulation of the synthesis of carotenoids precursors. In this study, bioinformatics analysis identified *SRE1* gene in the *X. dendrorhous* genome, and the predicted properties strongly suggest that this gene codes for the transcription factor Sre1 of the SREBP pathway that regulates the synthesis of sterols. The deduced protein has the characteristic topology and features of SREBP/Sre1 described in other organisms: (i) a conserved tyrosine residue at the bHLH motif, (ii) an N-terminal domain rich in serine and proline residues, and (iii) two potential transmembrane helixes after the bHLH motif. Moreover, by EMSA assays it was demonstrated that the purified bHLH protein from *X. dendrorhous* Sre1, binds to DNA fragments containing potential SRE sequences *in vitro*, and by ChIP-PCR bound the promoter region of target genes *in vivo*.

As in other organisms, the *SRE1* gene is not essential for the *X. dendrorhous* viability under standard laboratory culture conditions, but the yeast *sre1^-^* mutants are unable to grow in presence of azole compounds, such as clotrimazole. These compounds inhibit sterol synthesis as they interact with the catalytic site of the enzyme Cyp51/Erg11 favoring the production of cell-damaging sterols that affect the permeability and fluidity of the membrane, supporting that the identified gene is involved in the regulation of the synthesis of sterols as the RNA-seq data support (Table 3 and Figure 6). The same azole sensibility phenotype in *sre1^-^* mutants has been observed in other yeast such as *C. neoformans*, were the sensibility toward clotrimazole and itraconazole was higher in the mutant compared to the wild-type (Chang et al., 2007). Also, the more intense pigmentation of the strains cultivated with clotrimazole (Figure 5C) respect to the control condition (Figure 5B) could be explained by the sterol composition change generated by this compound that would activate the SREBP pathway, activating the mevalonate pathway and subsequently, carotenogenesis. In addition, the *X. dendrorhous* *sre1^-^* mutants did not grow in presence of chloride cobalt, which has been described as a hypoxia mimicking agent (Lee et al., 2007), suggesting that the *SRE1* gene product, also regulates the hypoxic response in *X. dendrorhous*; however, further analyses are required.

In a previous work, it was observed that by disrupting the *CYP61* gene in several *X. dendrorhous* strains, the production of ergosterol was blocked, but the production of other sterols and carotenoids was enhanced, suggesting a feedback regulatory mechanism in the sterol biosynthesis pathway and carotenogenesis of *X. dendrorhous* mediated by sterol composition (Loto et al., 2012). This is an interestingly aspect as little is known about the molecular mechanisms regulating carotenoid biosynthesis in *X. dendrorhous* and these observations positions the SREBP pathway as a good candidate to be involved in the regulation of carotenogenesis.

To our knowledge, there are no reports regarding the SREBP pathway in *X. dendrorhous*, and the results presented in this work, support that actually *X. dendrorhous* has an operative SREBP pathway. The *SRE1* gene was identified and interestingly, the *sre1^-^* mutation in a *cyp61^-^* genetic background, the mutant that overproduces carotenoids, leads to a wild-type carotenoid production demonstrating that the *SRE1* gene product is responsible, at least in part, for the carotenoid overproducing phenotype in the *cyp61^-^* mutants. In support to this statement, replacing the *SRE1* native gene by a version that encodes only Sre1N, strain *CBS.gSRE1N*, results in the same sterol and carotenoid overproducing phenotype as the *cyp61^-^* mutants. Results from strain *CBS.gSRE1N*, also demonstrate that the expression of the active form of Sre1 increases the production of both types of metabolites, sterols and carotenoids, suggesting that genes involved in their specific biosynthetic pathways or in the synthesis of their precursors, such as the mevalonate pathway, are Sre1 gene targets in *X. dendrorhous.*

Since the *sre1^-^* mutation significantly affected ergosterol levels in the wild-type strain, it is presumed that under the used culture conditions there are basal levels of Sre1N in *X. dendrorhous*, without requiring sterol composition changes for its activation. A low activation of Sre1 was reported in *S. pombe* and *C. neoformans* under standard culture conditions, this was determined by Western-blot assays in which low levels of the nuclear form of Sre1 (Sre1N) were observed in the wild-type strains (Hughes et al., 2005; Chang et al., 2007). Given the above and although there could be low levels of the active form of Sre1 in *X. dendrorhous*, these levels would be necessary and sufficient to regulate the sterol biosynthesis.

The results of RNA-seq between CBS.*sre1^-^* and the wild-type revealed a low transcriptional effect in response to the absence of Sre1, since only 11 genes decrease their expression in the mutant strain (Sre1-dependent genes), of which 8 participate in the mevalonate and sterol biosynthesis pathway: *HMGS*, *HMGR*, *ERG6*, *ERG7*, *ERG24* and *ERG25* and *CYP51*. These results would partially explain the decrease in sterol production of strain CBS.*sre1^-^* and therefore support the hypothesis that the SREBP pathway would be basally activated at basal levels of oxygen and sterols. Like *X. dendrorhous*, similar results were recently observed in *sre1^-^* mutants of *Penicillium digitatum* by RNA-seq, where without being subjected to any stimulus to activate the SREBP pathway, the expression of genes of the mevalonate and sterol biosynthesis pathway decreased (Ruan et al., 2017). No major differences were observed in the transcript levels of carotenogenic genes between CBS.*sre1-* and the wild-type, except for *crtR*, which is also involved in ergosterol biosynthesis. This indicates that Sre1 does not regulate the expression of exclusively carotenogenic genes but regulates carotenogenesis by regulating the expression of genes involved in the synthesis of carotenoid precursors at the mevalonate pathway and of *crtR*. Together, these results set Sre1, and other currently unknown components of the *X. dendrorhous* SREBP pathway, as potential targets to be manipulated in order to favor carotenoid production in *X. dendrorhous*.

Despite multiple attempts, we were not able to identify other components of SREBP pathway homologous to INSIG or SCAP (Bien and Espenshade, 2010), in the *X. dendrorhous* genome. However, similar scenarios have been described in other organisms. For example, even though *S. pombe* has an INSIG encoding gene homolog, its deletion did not affect Sre1 activation (Hughes et al., 2005). Rather, INSIG acts in the posttranslational regulation of HMGR (Burg and Espenshade, 2011). On the other hand, SCAP is less widely distributed than SREBP as it has been lost in several fungal species (Butler, 2013). Regarding this point, the *X. dendrorhous* Sre1 protein has a shorter C-terminal domain when compared to other fungal SREBPs and it lacks the DUF2014 domain present at the C-terminus in several SREBPs. It is possible that these differences at the C-terminal domain could be related to a SCAP-independent Sre1 activation mechanism and further studies are required. So, an interesting future challenge is to understand SREBP/Sre1 activation in organisms like *X. dendrorhous* that lack INSIG and SCAP.

## Conclusion

The bioinformatic, *in vitro* and *in vivo* DNA-binding properties, and the functional analyses performed in this work, support that the identified *X. dendrorhous* *SRE1* gene encodes a transcription factor that regulates the synthesis of isoprenoids in this yeast. Specifically, we demonstrate that mevalonate and ergosterol biosynthesis pathway genes are transcriptional targets of Sre1 using RNA-seq analysis. Moreover, results regarding sterols and carotenoids production in the *X. dendrorhous* strains, strongly suggest that the overproduction of carotenoids and sterols in strain CBS.cyp61- is due to the activation of the SREBP pathway and subsequent induction of HMGR and HMGS gene expression. Given that the sre1- mutation significantly decreased total sterol production in the wild-type strain (condition where Sre1 is not expected to be active), suggests basal Sre1N levels in the wild-type strain. To the best of our knowledge, this is the first report regarding the SREBP pathway in *X. dendrorhous* and also, the first report of the SREBP pathway effect over carotenogenesis.

## Data Availibility

RNA-Seq data available in the SRA database (PRJNA517352).

## Author Contributions

All authors contributed significantly to the work and all authors are in agreement with the content of the manuscript. MSG, SB, DS, AG, and MG performed the experiments. MF-L and SC performed the RNA-seq analysis. MB, VC, PE, and JA provided strategic input. MSG and JA wrote the manuscript.

## Conflict of Interest Statement

The authors declare that the research was conducted in the absence of any commercial or financial relationships that could be construed as a potential conflict of interest.

## Footnotes

1 http://www.qiagenbioinformatics.com
