# Supplementary material for: Sterol Regulatory Element-Binding Protein (Sre1) Promotes the Synthesis of Carotenoids and Sterols in Xanthophyllomyces dendrorhous
Source: Front Microbiol. 2019 Mar 29;10:586. doi: 10.3389/fmicb.2019.00586 (PMC6449425; doi:10.3389/fmicb.2019.00586)
Supplement: Supplementary file 1 [file Table_1.DOCX]

|  | **Primer name**  **Table S1.** Primers and probe designed and used in this work. | **SEQUENCE 5’ A 3’** | **Target and/or special features** |
| --- | --- | --- | --- |
|  | ***SRE1* gene deletion** | |  |
| **1** | Sre1.F | ATGGAAGACCTGTTCGAAG | *SRE1* gene |
| 2 | Sre1.1_R | TCAGCGTTTTGATGGGCC |  |
| 3 | Sre1-N_a.R | GGTGGCAGCGGTAGTGGTGGTG |  |
| 4 | Up2.Fw | GGGGAAGACTCGGAAGAGAA | *SRE1* promoter |
| 5 | gpdT.F | ACGGTTCTCTCCAAA | *gpd* terminator |
| 6 | Pef.R | TGAAGCTGTTCGAGA | *EF-1α* promoter |
| 7 | Sre_Del2_Out.R | TGTGACAGACGAGACCAAGAACAG | *SRE1* terminator |
| 8 | FLAG3X.Rv | TTTATCGTCGTCATCTTTGTA |  |
| 9 | Sre_Del1_Out.F | CCAAGACGAGAGAGGGACAAAACT | *SRE1* promoter |
| 10 | 8_Int3+HpaI.R | CTAGATCAGAGTTAACCGCACATCAATTCGAACAGC | *SRE1* promoter |
| 11 | Sre1_Down+HpaI.F | TTGATGTGCGGTTAACTCTGATCTAGTTTCCTTTTA | *SRE1* terminator |
| 12 | Sre_Del2.R | TCCAAGCGCTGATCGAAAGGTT | *SRE1* terminator |
| 13 | TEF.F | CGGCTCATCAGCCGACAGTTCA | *EF-1α* promoter |
| 14 | GPD.R | ATGAGAGATGACGGAGATGAT | *gpd* terminator |
|  | **Heterologous complementation in *S. pombe*** | |  |
|  | Sre1.F+HpaI | TTTTTTGTTAACATGGAAGACCTGTTCGAAG | *SRE1* gene |
|  | Sre1_Nc+SphI.R | TTTTTTGCATCGTCAGGGGTGGTGGCGGTGGAGGGGG | *SRE1* gene |
|  | ***DNA assembler*** | |  |
| 15 | A.Fw | TATCATGCGTCAATCGTATGTGAATGCTGGTCGCTATACTGGCATTAAGCGCGGCGGGTG | pBS |
| 16 | A.Rv | AGTCCGTGGAATTAATTCTCATCTTTGACAGCTTATCATCGATAAGTGCGCGGAACCCCT | pBS |
| 17 | B.Fw | GAATGTATTTAGAAAAATAAACAAATAGGGGTTCCGCGCACTTATCGATGATAAGCTGTC | pYES2 |
| 18 | B.Rv | ACGTCCTTCGTAGTTTTGTCCCTCTCTCGTCTTGGGTTAACCATTGCGAATACCGCTTCC | pYES2 |
| 19 | C.Fw | TACTTTTGAGCAATGTTTGTGGAAGCGGTATTCGCAATGGTTAACCCAAGACGAGAGAGG | *SRE1* promoter |
| 20 | C_2.Rv | ATATCATGATCTTTATAATCACCGTCATGGTCTTTGTAGTCCATTGTGTGTGTGTGTGGG | *SRE1* promoter |
| 21 | D_2.Fw | CCATAGATAAGCACACCCACACCCACACACACACACAATGGACTACAAAGACCATGACGG | pFlagTEM1 |
| 22 | D_2.Rv | TCGAACCGGAATGGATCTTTTTCTTCGAACAGGTCTTCCATTTTATCGTCGTCATCTTTG | pFlagTEM1 |
| 23 | E_Nt (stop).Fw | AGTGACGGCCACCGTCACCACCACTACCGCTGCCACCTGATCTGATCTAGTTTCCTTTTA | *SRE1* terminator |
| 24 | E.Rv | CAAAGAGCTTGTGTCGGATGAACTGTCGGCTGATGAGCCGATTGGGCAGACGGAAGCATG | *SRE1* terminator |
| 25 | F.Fw | TACTGTCTCGATCTGCTTTCATCTTTTCATGCTTCCGTCTGCCCAATCGGCTCATCAGCC | pIR-*zeo* |
| 26 | F.Rv | AAAGACAAAAAGGAAGAGAAAAATAAAAGGAAACTAGATCAGAATCATGAGAGATGACGG | pIR-*zeo* |
| 27 | G.Fw | TCACATCTGTTGACCATCACCATCATCTCCGTCATCTCTCATGATTCTGATCTAGTTTCC | *SRE1* terminator |
| 27 | G.Rv | GTCAAGACTGTCAAGGAGGGTATTCTGGGCCTCCATGTCGTTAACTCCAAGCGCTGATCG | *SRE1* terminator |
| 28 | H.Fw | TCAATCTATCATATGTAAACCTTTCGATCAGCGCTTGGAGTTAACGACATGGAGGCCCAG | pFA6a |
| 29 | H.Rv | GCGGTCACGCTGCGCGTAACCACCACACCCGCCGCGCTTAATGCCAGTATAGCGACCAGC | pFA6a |
| 30 | SRE1_ATG.Fw | ATAAAGATCATGATATCGACTACAAAGATGACGACGATAAAATGGAAGACCTGTTCGAAG | *SRE1* gene |
| 31 | SRE1-N(stop)+term.Rv | AAAAAGGAAGAGAAAAATAAAAGGAAACTAGATCAGATCAGGTGGCAGCGGTAGTGGTGG | *SRE1* gene |
|  |  |  |  |
|  | **EMSA Assay Biotinylated probe:** |  |  |
| 32 | pHMGS SRE1+SRE2.Fw | CCATCGTCACTCTCGTCTCCTGACCCGTTGGGGCTATGTAACACCACACGTGACTCCACT | *HMGS* promoter |
| 33 | pHMGS SRE1+SRE2.Rv | AGTGGAGTCACGTGTGGTGTTACATAGCCCCAACGGGTCAGGAGACGAGAGTGACGATGG | *HMGS* promoter |
| 34 | pHMGS SRE1*+SRE2.Fw | CCATCGTCACTCTTTATTCTTTTTCCGTTGGGGCTATGTAACACCACACGTGACTCCACT | *HMGS* promoter |
| 35 | pHMGS SRE1*+SRE2.Rv | AGTGGAGTCACGTGTGGTGTTACATAGCCCCAACGGAAAAAGAATAAAGAGTGACGATGG | *HMGS* promoter |
| 36 | pHMGS SRE1+SRE2*.Fw | CCATCGTCACTCTCGTCTCCTGACCCGTTGGGGCTATAATTCATTTTACGTGACTCCACT | *HMGS* promoter |
| 37 | pHMGS SRE1+SRE2*.Rv | AGTGGAGTCACGTAAAATGAATTATAGCCCCAACGGGTCAGGAGACGAGAGTGACGATGG | *HMGS* promoter |
| 38 | pHMGS SRE1*+SRE2*.Fw | CCATCGTCACTCTTTATTCTTTTTCCGTTGGGGCTATAATTCATTTTACGTGACTCCACT | *HMGS* promoter |
| 39 | pHMGS SRE1*+SRE2*.Rv | AGTGGAGTCACGTAAAATGAATTATAGCCCCAACGGAAAAAGAATAAAGAGTGACGATGG | *HMGS* promoter |
|  | **RT-qPCR analysis:** | |  |
| 40 | mactF-RT | CCGCCCTCGTGATTGATAAC | *ACT* gene |
| 41 | mactR-RT | TCACCAACGTAGGAGTCCTT | *ACT* gene |
| 42 | HMGR_Real_2_F | GGCCGATCGCTATACATCCGTTT | *HMGR* gene |
| 43 | HMGR_Real_2_R | ATGCAGTTGATGGCAGAAGGCT | *HMGR* gene |
| 44 | hmgS.RT2.F | AAGCAGGTTGAGCCTGGAATGA | *HMGS* gene |
| 45 | hmgS.RT2.R | AAAGCGGTTGAGCTCTTGACCT | *HMGS* gene |
|  | **ChIP – PCR:** |  |  |
| 46 | prom.hs.Fw | CGAGAGTATCGTCCCATCTAT | *HMGS* promoter |
| 47 | prom.hs.Rv | AACTGGATATCTAGATTGAGA | *HMGS* promoter |
| 48 | prom.Hr.F1 | CCAGCTGTGACCTCACGTGACTA | *HMGR* promoter |
| 49 | prom.Hr.R | CAACTGAAAGTACGTCAAGGT | *HMGR* promoter |
| 50 | Prom.grg2.F | GCGTGAAGAAGTATCTGGTTC | *GRG2* promoter |
| 51 | Prom.grg2.R | GGATAGGTGGGTGGATGAAG | *GRG2* promoter |
