# Supplementary material for: Sterol Regulatory Element-Binding Protein (Sre1) Promotes the Synthesis of Carotenoids and Sterols in Xanthophyllomyces dendrorhous
Source: Front Microbiol. 2019 Mar 29;10:586. doi: 10.3389/fmicb.2019.00586 (PMC6449425; doi:10.3389/fmicb.2019.00586)
Supplement: Supplementary file 2 [file Table_2.DOCX]

**Table S2.** Potential Sre1 binding sequences (SRE) identified for TFBIND and JASPAR programs in the same position.

| **Gene** | **Sequence (5´ - 3´)** | **Position 5´ - 3´^a^** | | |
| --- | --- | --- | --- | --- |
| **MVA Pathway** | |  |  |  |
| *ERG10* | GATCAGGCGAC | - 600 | ̶̶ | -590 |
| *HMGR* | CCTCACGTGAC | -770 | ̶̶ | -760 |
| *HMGS* | TGTAACACCAC | -244 | ̶̶ | -234 |
|  | CCACACGTGAC | -237 | ̶̶ | -227 |
| *PMK* | GATCACCTCTT | -51 | ̶̶ | -41 |
|  | GTTCACGTGAT | -96 | ̶̶ | -86 |
|  | |  |  |  |
| **Ergosterol Biosynthesis** | |  |  |  |
| *CYP61* | CATCACCAGAG | -529 | ̶̶ | -519 |
| *ERG1* | TTTCACCCAAC | -270 | ̶̶ | -260 |
|  | GAACACTCCAC | 245- | ̶̶ | -235 |
| *ERG2* | TCTCACGTGAC | -119 | ̶̶ | -109 |
| *ERG6* | AGGCACGTGCC | -225 | ̶̶ | -215 |
| *ERG7* | ATCACCACAG | -219 | ̶̶ | -210 |
| *ERG25* | CATCAGCCCAC | -992 | ̶̶ | -982 |
|  | |  |  |  |
| **Carotenoids Biosynthesis** | |  |  |  |
| *crtR* | TATCTCCTCAC | -68 | ̶̶ | -58 |
|  | GCTCACGTGAC | -345 | ̶̶ | -335 |
| *crtYB* | GATCACGTGAG | -383 | ̶̶ | -373 |
| *FPS* | GATCATCTCAC | -895 | ̶̶ | -885 |

^a^ All sequences were identified in sense strand
