# Supplementary figures and images for: Sterol Regulatory Element-Binding Protein (Sre1) Promotes the Synthesis of Carotenoids and Sterols in Xanthophyllomyces dendrorhous
Source: Front Microbiol. 2019 Mar 29;10:586. doi: 10.3389/fmicb.2019.00586 (PMC6449425; doi:10.3389/fmicb.2019.00586)

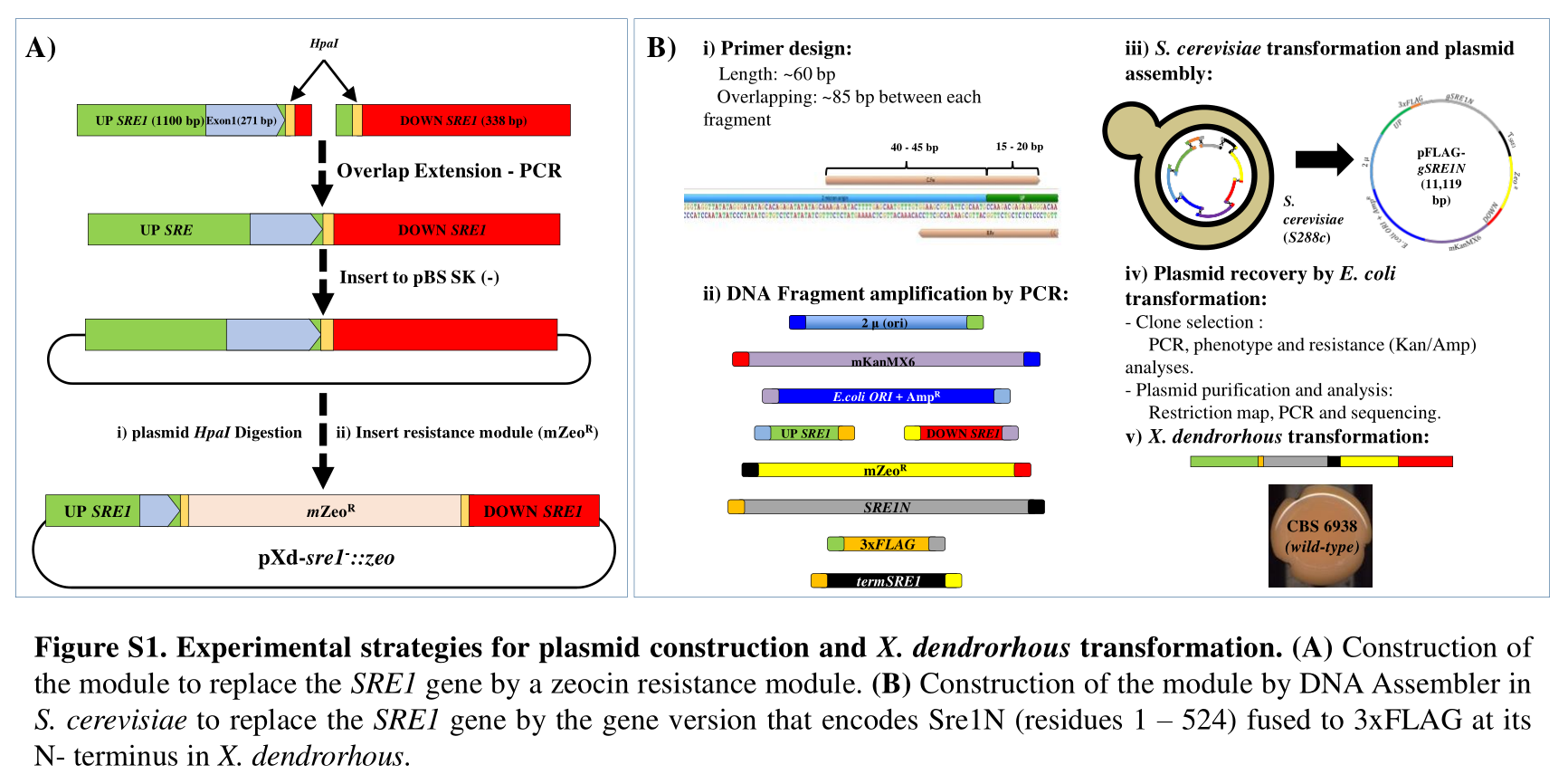

Supplement: Supplementary file 3 [file Image_1.TIFF]

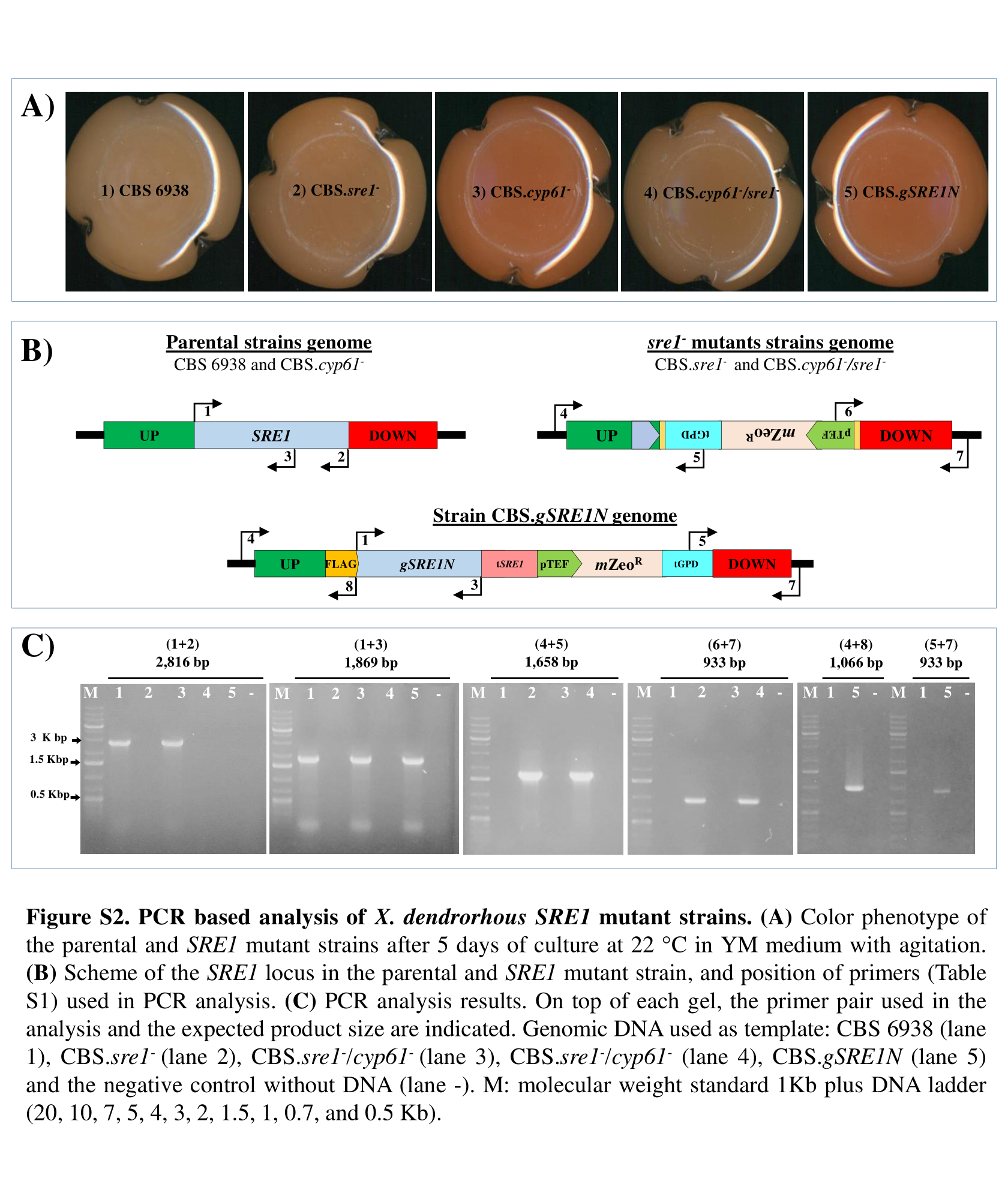

Supplement: Supplementary file 4 [file Image_2.TIFF]

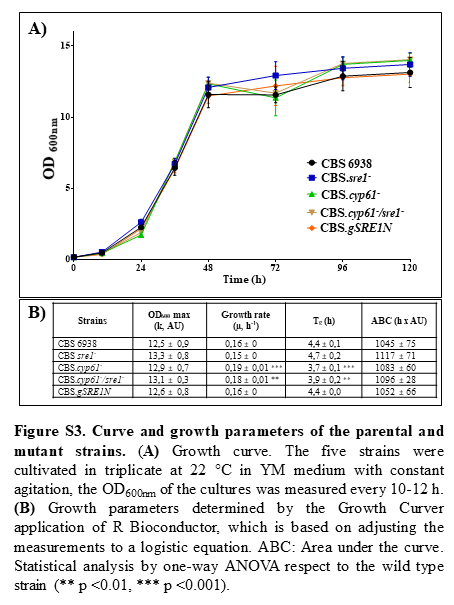

Supplement: Supplementary file 5 [file Image_3.TIF]
